# Five-Factor Model personality profiles of drug users

**DOI:** 10.1186/1471-244X-8-22

**Published:** 2008-04-11

**Authors:** Antonio Terracciano, Corinna E Löckenhoff, Rosa M Crum, O Joseph Bienvenu, Paul T Costa

**Affiliations:** 1 National Institute on Aging, NIH, DHHS, Baltimore, USA; 2 Johns Hopkins Bloomberg School of Public Health, Baltimore, USA

## Abstract

**Background:**

Personality traits are considered risk factors for drug use, and, in turn, the psychoactive substances impact individuals' traits. Furthermore, there is increasing interest in developing treatment approaches that match an individual's personality profile. To advance our knowledge of the role of individual differences in drug use, the present study compares the personality profile of tobacco, marijuana, cocaine, and heroin users and non-users using the wide spectrum Five-Factor Model (FFM) of personality in a diverse community sample.

**Method:**

Participants (*N *= 1,102; mean age = 57) were part of the Epidemiologic Catchment Area (ECA) program in Baltimore, MD, USA. The sample was drawn from a community with a wide range of socio-economic conditions. Personality traits were assessed with the Revised NEO Personality Inventory (NEO-PI-R), and psychoactive substance use was assessed with systematic interview.

**Results:**

Compared to never smokers, current cigarette smokers score lower on Conscientiousness and higher on Neuroticism. Similar, but more extreme, is the profile of cocaine/heroin users, which score very high on Neuroticism, especially Vulnerability, and very low on Conscientiousness, particularly Competence, Achievement-Striving, and Deliberation. By contrast, marijuana users score high on Openness to Experience, average on Neuroticism, but low on Agreeableness and Conscientiousness.

**Conclusion:**

In addition to confirming high levels of negative affect and impulsive traits, this study highlights the links between drug use and low Conscientiousness. These links provide insight into the etiology of drug use and have implications for public health interventions.

## Background

Drug use is related to adverse health and social outcomes [1]. Cigarette smoking is a leading cause of preventable disability and death in the U.S. and around the world [2], increasing the risk of cancer, cardiovascular disease, respiratory and other health problems [3,4]. The use of other psychoactive substances, most of them illicit drugs, is also associated with massive social cost beyond the damage to the individual users, affecting health care, law enforcement, and legal systems [1,5].

The high individual and social costs of drug use highlight the need to study factors related to such behaviors. Even if personality differences between drug users and non-users are generally small, these effects can have important clinical implications due to the large number of people involved. Research on the correlates of drug addiction provides insights for understanding etiology and inform prevention policies and cessation programs. For example, from a psychiatric perspective, a number of studies have documented the high comorbidity of drug use with other mental disorders [6-10], which indicate that mood, anxiety, and personality disorders need to be considered by drug treatment specialists to achieve successful intervention. The present study contributes to this line of research by examining the personality traits associated with current and lifetime drug use in an economically-diverse community sample. Specifically, we attempt to replicate previously reported associations among personality traits and smoking status and extend the analyses to users of marijuana, heroin, and cocaine. Comparing personality profiles, we examine similarities and differences in traits associated with a variety of drugs used. Although the high rate of multiple drug use complicates comparisons across substances, results may point to specific traits that underlie the use of a specific drug as well as common factors across different types of drug use.

The present study adopts the well-validated Five-Factor Model of personality [11] which comprehensively covers the five major traits that define human personality across cultures [12,13]: Neuroticism (N), the tendency to experience negative emotions such as anxiety and depression; Extraversion (E), the tendency to be sociable, warm, active, assertive, cheerful, and in search of stimulation; Openness to Experience (O), the tendency to be imaginative, creative, unconventional, emotionally and artistically sensitive; Agreeableness (A), the dimension of interpersonal relations, characterized by altruism, trust, modesty, and cooperativeness; and Conscientiousness (C), a tendency to be organized, strong-willed, persistent, reliable, and a follower of rules and ethical principles. Each of these factors is hierarchically defined by specific facets, which can provide a more in-depth description of drug users' personalities.

In previous studies, cigarette smokers were found to score high on facets related to impulsivity and Neuroticism, and low on Agreeableness and Conscientiousness [14-16]. However, in European and Asian studies and some older US studies, smokers were also found to score high on Extraversion [16-18]. Compared to cigarette smoking, there are fewer studies on the personality correlates of illegal drug use, and these are based on smaller sample sizes and a variety of personality measures. A meta-analysis [19] examined personality correlates of marijuana use categorizing traits into "negative affect" (e.g., depression, anxiety), "emotionality" (e.g., extraversion, social disinhibition), and "unconventionality" (e.g., tolerance of deviance, non-religiosity). These measures map loosely onto Neuroticism, Extraversion, and Openness, respectively. Results suggested that marijuana use was related to high levels of unconventionality, and only weakly to emotionality and negative affect. Another meta-analysis [20] examined the role of a wide range of Conscientiousness-related measures on health risk behaviors. Across studies, a consistent association was found between marijuana use (as well as other drug use) and low scores on Conscientiousness-related traits. Cocaine users are characterized by high scores on Neuroticism-related traits [21,22], such as depression and impulsivity [23,24], as well as Psychoticism [21,22], a trait related to low Agreeableness and low Conscientiousness. Finally, studies of heroin users consistently depict them as high on Neuroticism [25-28]. Many studies show an association of heroin use with high Extraversion and high Psychoticism, but this association appears to be less robust [26-28]. Inconsistencies in the association of personality and drug use are due to several factors, such as differences in the personality measures used, inadequate sample sizes, and socio-cultural differences. Most studies use measures that capture only a subset of relevant personality traits, and rarely assess all five major factors and their facets. Studies are also hampered by reliance on small convenience samples. This is particularly true for studies on the use of illegal drugs. Further, with few exceptions, studies have focused on a single substance at a time, making it difficult to detect common patterns across a range of different drugs. This study extends previous research by examining multiple types of drug use in a large population-based sample while utilizing a well-validated and comprehensive measure of personality that captures both global factors and specific facets of personality.

## Method

### Participants

Participants were drawn from the East Baltimore Epidemiologic Catchment Area Study (Baltimore ECA) [29], a multidisciplinary study which is based on a probability sample of 3,481 East Baltimore residents who were initially interviewed in 1981 and followed up in 1992–98 and in 2004–05. Personality traits were assessed at the two most recent waves. To maximize the sample size, cross-sectional analyses were conducted on the last valid personality assessments (*N *= 1,102), 80% of which were obtained in 2004–05. At the time of the personality assessments, age ranged from 30 to 94 years (M = 56.6; *SD *= 12.4), and participants had an average of 12.5 years of education (*SD *= 2.6). About 62% of the sample was female; 63% were White/Non-Hispanic, 34% were Black/Non-Hispanic, and 3% other or unknown ethnic group. To screen out cognitively impaired individuals, participants with Mini Mental State Scores [30] below the cut-off value of 23 were excluded.

As could be expected, participants who completed the personality measure at follow-up were those who in 1981 were younger (35 vs. 54 years old; *p *< .01) and more educated (12.5 vs. 9.7 years of education; *p *< .01), as compared to those who did not complete the personality assessment (because of mortality, sample loss, or subject refusal). There were no significant differences in the proportion of males and females or ethnic groups (Fisher's exact test: *p *> .05).

### Drug use assessment and prevalence

Trained interviewers asked questions about substance use after participants signed the informed consent approved by the ethics committee. The form assured the confidentiality of the answers, and that those were used for research purposes only. Participation was voluntary, and subjects could withdraw at any time. Subjects received $20 after the interview.

Cigarette smoking status was determined by responses to interview questions, asking participants whether they had ever smoked tobacco cigarettes and when they smoked their last cigarette. Of the 1,088 participants without missing data, we classified "never smokers" as those who never smoked (*n *= 341), "former smokers" as those who smoked but not in the last seven days (*n *= 429), and current smokers as those who smoked in the last seven days (*n *= 318). Use ranged from fewer than 10 cigarettes (40%) to over 20 cigarettes a day (16%), with a majority smoking between 11 and 20 cigarettes a day (44%).

Marijuana use was determined by responses to questions on whether participants had ever used either marijuana or hashish, even once, and when they last used marijuana or hashish. Of the 1044 individuals without missing data, about 55% of the sample responded that they never tried marijuana or hashish (*n *= 576), 36% used it but not in the past-year (*n *= 380), and 8% did use it in the last-year (*n *= 88). Among the past-year current users, about 20% used marijuana daily or almost daily, about 45% used it from once or twice a week to once or twice a month, and about 35% used it between once and eleven times a year.

Cocaine use was determined by responses to questions on whether participants had ever used cocaine (including all forms of cocaine, such as powder, "crack," freebase, and coca paste) even once, and when they last used any form of cocaine. Of the 1094 individuals without missing data, about 82% had never tried cocaine (*n *= 896), about 16% used it but not in the past-year (*n *= 170), and about 3% did use it in the last-year (*n *= 28). Among the past-year current users, about 14% used cocaine daily or almost daily, about 43% used it from once or twice a week to once or twice a month, and about 43% used it between once and eleven times a year.

Heroin use was determined by responses to questions on whether participants had ever used heroin, even once, and when they last used it. Of the 1094 individuals without missing data, about 93% had never tried heroin (*n *= 1023), about 6% used it but not in the past-year (*n *= 62), and about 1% used it in the last-year (*n *= 9). Of the nine past-year current users, three individuals used heroin daily or almost daily, two used it from once or twice a week to once or twice a month, and four used it between once and eleven times a year. Given the small number of current heroin users, and given that eight out of nine current heroin users were also current cocaine users, and 87% of the former heroin users were also former or current cocaine users, we examined the association of personality traits with cocaine *or *heroin use. As expected in a population-based sample, we also found substantial overlap among the other drugs used. Of the former and current smokers, 52% had also used marijuana, 21% cocaine, and 8% heroin. About 78% of current and former marijuana users had also smoked cigarettes, 40% had used cocaine, and 15% heroin. About 82% of current and former heroin/cocaine users had also smoked cigarettes and 98% had used marijuana. Demographic information by drug type is given in Table 1: Across substances, current users were younger; males were more likely to use marijuana and cocaine/heroin, but there were no sex differences in cigarette smoking; African-Americans were more likely to use substances; and low education was associated with higher use of substances, with the exception of marijuana.

**Table 1:** Demographic statistics by drug type.

Group	N	Age	Female	African-American	Education
Never smoker	341	58	64%	30%	12.9
Former smoker	429	59	60%	30%	12.6
Current smoker	318	52	62%	45%	11.9
					
Marijuana: never-user	576	62	69%	30%	12.1
Marijuana: former-user	380	50	53%	37%	13.0
Marijuana: current-user	88	48	44%	47%	12.4
					
Cocaine/heroin: never-user	887	59	65%	32%	12.4
Cocaine/heroin: former-user	178	48	46%	39%	12.9
Cocaine/heroin: current-user	29	46	48%	59%	11.9
					
Total	1102	57	62%	34%	12.5

*Note*. Mean age and education expressed in years. For age, ethnicity, and education there were significant differences between never, former, and current users of cigarettes, marijuana, and cocaine/heroin. Sex differences were seen for marijuana and cocaine/heroin users, but not for cigarette smoking.

### Personality assessment

Participants completed the self-report questionnaire at home or at a participating institution. The Revised NEO Personality Inventory [NEO-PI-R, [31]] consists of 240 items answered on a five-point Likert format ranging from *strongly disagree *to s*trongly agree*. The NEO-PI-R assesses 30 facets, six for each dimension of the FFM (see Table 2 for a listing of the 30 facet scales). Raw scores were standardized as *T-*scores (*M *= 50, *SD *= 10) using combined-sex adult norms reported in the *Manual *[31]. The NEO-PI-R has been translated into several languages and used in more than 50 cultures [12]. Evidence of convergent and discriminant validity is presented in the *Manual *[31], and a large literature demonstrates cross-observer agreement and prediction of external criteria such as psychological well-being, health risk behaviors, educational and occupational achievements, coping mechanisms, and longevity [31,32]. In a previous study [33] we tested the validity of personality assessment in the ECA sample and found adequate alpha reliabilities, retest-stability, and factor structure of the NEO-PI-R scales.

### Statistical analyses

All analyses were performed using SPSS 13.0 [34]. For each drug, we performed a MANCOVA with user status as the independent variable, personality factors and facets as the dependent variables, and age, sex, education, and ethnicity as covariates. Post-hoc comparisons among never, former, and current users groups were based on LSD estimates. The assumption of homogeneity of variance was tested using the Levene statistic, and no large violations were found. Effect sizes were estimated using partial η^2^. According to Cohen [35], η^2 ^values of 0.0099, 0.0588 and 0.1379 correspond to small, medium and large effect sizes, respectively.

## Results

### Personality traits and smoking status

**Figure 1:**
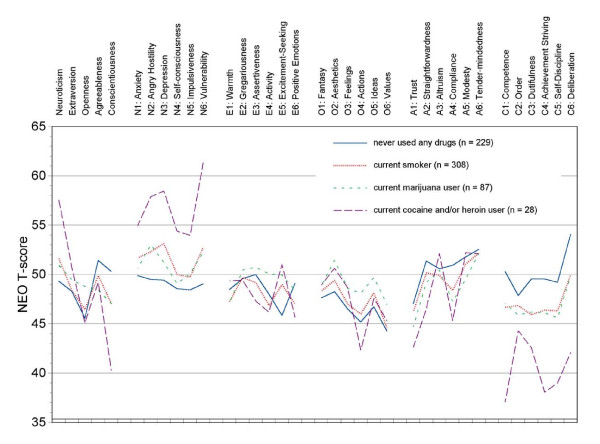
**NEO-PI-R profile of current users of tobacco, marijuana, and cocaine/heroin and never users of those substances**. Plots show estimated marginal means after controlling for age, sex, ethnicity, and education.

Multivariate analyses of covariance controlling for demographic variables indicated significant personality differences among smoking status groups (see Table 2). Compared to never smokers, current smokers scored higher on Neuroticism and lower on Conscientiousness. Current smokers scored lower on Agreeableness, but this difference was not significant after controlling for demographic variables. Former smokers scored intermediate on Neuroticism and Conscientiousness. At the facet level, current smokers were characterized by traits related to the construct of impulsivity, (i.e., N5:Impulsiveness, E5:Excitement-Seeking, C5:Self-Discipline, and C6:Deliberation) [36]. Smokers as a group were also high on N3:Depression, N6:Vulnerability, and low in A4:Compliance, C1:Competence, and C3:Dutifulness. Although the magnitude of the effects is small, the differences among groups are consistent with the findings from previous studies [14]. Figure 1 plots the full profile of current smokers against current users of marijuana and cocaine/heroin, as well as never user of these substances. The Figure presents estimated marginal means after partialling out the demographic covariates. Former users are excluded.

**Table 2:** Mean personality traits for never, former, and current cigarette smokers.

NEO-PI-R scales	Never-smokers (*n *= 341)	Former-smokers (*n *= 429)	Current-smokers (*n *= 318)	*F*	Partial η^2^
Neuroticism	48.9 (.51)^a^	50.7 (.46)^b^	51.6 (.55)^b^	6.9**	.013
Extraversion	48.8 (.44)	49.3 (.40)	48.4 (.48)	1.0	.002
Openness	45.6 (.44)	46.4 (.39)	46.5 (.47)	1.2	.002
Agreeableness	50.9 (.49)	50.9 (.44)	49.9 (.52)	1.3	.002
Conscientiousness	49.4 (.52)^a^	49.0 (.46)^a^	47.0 (.55)^b^	5.5**	.010
					
N1: Anxiety	49.7 (.46)^a^	51.0 (.41)^b^	51.7 (.50)^b^	4.7**	.009
N2: Angry Hostility	49.5 (.50)^a^	50.7 (.45)^a^	52.3 (.54)^b^	6.9**	.013
N3: Depression	49.3 (.57)^a^	50.8 (.51)^a^	53.1 (.61)^b^	10.0**	.019
N4: Self-consciousness	48.4 (.52)^a^	50.1 (.46)^b^	50.0 (.56)^b^	3.6*	.007
N5: Impulsiveness	48.3 (.48)^a^	49.8 (.43)^b^	49.7 (.52)^b^	3.4*	.007
N6: Vulnerability	49.3 (.58)^a^	50.4 (.52)^a^	52.7 (.63)^b^	7.9**	.015
E1: Warmth	48.9 (.52)	48.4 (.46)	47.2 (.56)	2.4	.005
E2: Gregariousness	50.5 (.50)	51.0 (.45)	49.7 (.54)	1.7	.003
E3: Assertiveness	50.1 (.51)	49.6 (.46)	49.2 (.55)	0.7	.001
E4: Activity	48.1 (.49)	47.3 (.43)	46.9 (.52)	1.5	.003
E5: Excitement-Seeking	46.9 (.47)^a^	47.7 (.42)^a^	49.0 (.50)^b^	4.4*	.008
E6: Positive Emotions	48.9 (.51)^a^	48.9 (.46)^a^	47.0 (.55)^b^	3.9*	.007
O1: Fantasy	47.8 (.44)	48.5 (.39)	48.3 (.47)	0.7	.001
O2: Aesthetics	48.3 (.48)	49.4 (.43)	49.4 (.52)	1.7	.003
O3: Feelings	46.7 (.46)	47.6 (.41)	47.0 (.50)	1.2	.002
O4: Actions	45.2 (.50)	46.1 (.45)	46.0 (.54)	1.0	.002
O5: Ideas	46.5 (.48)	46.9 (.43)	48.1 (.52)	2.7	.005
O6: Values	45.0 (.45)	45.6 (.40)	44.6 (.48)	1.4	.003
A1: Trust	47.2 (.53)	46.6 (.47)	46.2 (.57)	0.9	.002
A2: Straightforwardness	50.7 (.50)	51.0 (.44)	50.2 (.53)	0.7	.001
A3: Altruism	50.2 (.52)^a^	51.6 (.46)^b^	49.9 (.55)^a^	3.5*	.007
A4: Compliance	50.5 (.55)^a^	48.8 (.49)^b^	48.4 (.59)^b^	3.8*	.007
A5: Modesty	50.9 (.50)	51.5 (.45)	51.1 (.54)	0.3	.001
A6: Tender-mindedness	52.0 (.50)	52.4 (.44)	52.2 (.53)	0.2	.000
C1: Competence	50.0 (.56)^a^	49.9 (.50)^a^	46.7 (.60)^b^	10.2**	.019
C2: Order	47.5 (.47)	47.0 (.42)	46.8 (.51)	0.5	.001
C3: Dutifulness	49.0 (.51)^a^	48.5 (.45)^a^	45.9 (.55)^b^	9.0**	.017
C4: Achievement Striving	49.0 (.55)^a^	48.4 (.49)^a^	46.4 (.58)^b^	5.4**	.010
C5: Self-Discipline	48.6 (.55)^a^	47.7 (.49)^a^	46.3 (.59)^b^	3.8*	.007
C6: Deliberation	53.5 (.53)^a^	52.6 (.48)^a^	50.0 (.57)^b^	10.1**	.019

*Note*. Estimated marginal means (Standard Errors), statistical tests, and effect sizes computed after controlling for age, sex, ethnicity (black, white), and education (*n *= 1058).

Means with different superscripts differ significantly at *p *< .05.

* *p *< .05; ** *p *< .01

### Personality traits and marijuana use

Compared to never users, current marijuana users scored higher on Openness and lower on Agreeableness and Conscientiousness (see Table 3). Former users scored intermediate on these three factors. On the facet level, current marijuana users scored higher on four facets of Openness, particularly Openness to O6:Values and O5:Ideas, and lower on five facets of Conscientiousness, particularly C3:Dutifulness and C6:Deliberation. Other interesting effects for current marijuana users were the high scores on N2:Angry Hostility, N6:Vulnerability, E4:Activity, and E5:Excitement Seeking, and the low scores on A4:Compliance, as compared to never users. As depicted in Figure 1, the profile of current marijuana users is similar to the pattern found among current smokers, especially for the facets of Conscientiousness.

**Table 3:** Mean personality traits for never, non-current, and current marijuana users.

NEO-PI-R scales	Never-user (*n *= 576)	Former user (*n *= 380)	Current user (*n *= 88)	*F*	Partial η^2^
Neuroticism	50.0 (.42)	50.4 (.50)	50.9 (1.01)	0.4	.001
Extraversion	49.0 (.37)	48.5 (.44)	49.4 (.88)	0.7	.001
Openness	45.5 (.37)^a^	46.1 (.44)^a^	48.8 (.88)^b^	5.5**	.011
Agreeableness	51.6 (.40)^a^	49.6 (.49)^b^	48.4 (.98)^b^	6.3**	.012
Conscientiousness	49.7 (.43)^a^	47.0 (.51)^b^	47.0 (1.03)^b^	8.0**	.016
					
N1: Anxiety	50.5 (.38)	50.8 (.46)	50.6 (.92)	0.1	.000
N2: Angry Hostility	50.0 (.41)^a^	51.2 (.50)^ab^	53.0 (1.00)^b^	3.9*	.008
N3: Depression	50.4 (.47)	51.4 (.57)	51.2 (1.14)	0.7	.001
N4: Self-consciousness	49.2 (.43)	50.0 (.51)	49.1 (1.03)	0.7	.001
N5: Impulsiveness	48.7 (.40)	49.7 (.48)	50.1 (.96)	1.4	.003
N6: Vulnerability	49.6 (.47)^a^	51.5 (.57)^b^	52.2 (1.15)^b^	3.7*	.007
E1: Warmth	49.1 (.43)^a^	47.1 (.52)^b^	47.1 (1.03)^ab^	4.2*	.008
E2: Gregariousness	50.8 (.41)	50.1 (.50)	50.4 (1.00)	0.5	.001
E3: Assertiveness	49.7 (.42)	49.4 (.51)	50.7 (1.03)	0.7	.001
E4: Activity	47.4 (.40)^a^	46.9 (.48)^a^	50.1 (.97)^b^	4.6*	.009
E5: Excitement-Seeking	47.1 (.39)^a^	48.5 (.47)^b^	49.9 (.94)^b^	4.3*	.009
E6: Positive Emotions	49.0 (.42)	47.4 (.51)	48.4 (1.02)	2.3	.005
O1: Fantasy	47.9 (.36)	48.5 (.44)	48.9 (.87)	0.8	.001
O2: Aesthetics	48.5 (.40)^a^	49.0 (.48)^a^	51.4 (.96)^b^	3.9*	.008
O3: Feelings	46.6 (.38)	47.3 (.46)	48.6 (.92)	1.9	.004
O4: Actions	45.2 (.42)^a^	45.9 (.50)^a^	48.1 (1.01)^b^	3.2*	.006
O5: Ideas	47.2 (.40)^a^	46.2 (.49)^a^	49.7 (.97)^b^	5.7**	.011
O6: Values	44.0 (.37)^a^	46.1 (.44)^b^	46.9 (.89)^b^	7.9**	.015
A1: Trust	47.3 (.43)	46.5 (.52)	44.7 (1.04)	2.7	.005
A2: Straightforwardness	51.4 (.41)	50.0 (.49)	49.2 (.99)	2.9	.006
A3: Altruism	51.4 (.42)^a^	49.4 (.51)^b^	50.4 (1.03)^ab^	3.8*	.008
A4: Compliance	50.4 (.45)^a^	48.2 (.55)^b^	47.2 (1.10)^b^	5.4**	.011
A5: Modesty	51.9 (.42)	50.7 (.50)	49.5 (1.01)	2.8	.006
A6: Tender-mindedness	52.5 (.41)	51.6 (.50)	52.2 (.99)	1.0	.002
C1: Competence	50.0 (.47)^a^	47.7 (.56)^b^	47.1 (1.13)^b^	5.0**	.010
C2: Order	47.6 (.39)	46.5 (.48)	45.9 (.95)	2.1	.004
C3: Dutifulness	49.2 (.42)^a^	46.2 (.51)^b^	46.2 (1.01)^b^	9.8**	.019
C4: Achievement Striving	49.0 (.45)^a^	46.6 (.55)^b^	46.1 (1.09)^b^	6.0**	.012
C5: Self-Discipline	48.7 (.46)^a^	46.5 (.55)^b^	45.6 (1.10)^b^	5.3**	.010
C6: Deliberation	53.2 (.44)^a^	50.9 (.54)^b^	49.9 (1.07)^b^	6.6**	.013

*Note*. Estimated marginal means (Standard Errors), statistical tests, and effect sizes computed after controlling for age, sex, ethnicity (black, white), and education (*n *= 1013).

Means with different superscripts differ significantly at *p *< .05.

* *p *< .05; ** *p *< .01

### Personality traits and cocaine/heroin use

Compared to never users, current cocaine/heroin users scored higher on Neuroticism and lower on Conscientiousness (see Table 4). Former cocaine/heroin users scored lower on Conscientiousness, but did not differ from never-users on Neuroticism. On the facet level, current users scored high on all facets of Neuroticism, with large effect sizes (difference larger than one *SD*) on N6:Vulnerability, high on E5:Excitement Seeking, low on A1:Trust, A2:Straightforwardness, and A4:Compliance, and very low on all facets of Conscientiousness, with differences larger than one *SD *on C1:Competence, C4:Achievement Striving, and C6:Deliberation. The profile of cocaine/heroin current users is illustrated in Figure 1, which resembles the pattern seen for current smokers, but the profile of cocaine/heroin users is more extreme. Additional analyses indicated that although the few individuals (*n *= 9) who used both cocaine and heroin had the most extreme profile, the individuals who were current users of cocaine but not heroin also scored significantly higher on Neuroticism and lower on Conscientiousness than never users.

**Table 4:** Mean personality traits for never, non-current, and current cocaine/heroin users.

NEO-PI-R scales	Never-user (*n *= 887)	Former user (*n *= 178)	Current user (*n *= 29)	*F*	Partial η^2^
Neuroticism	50.3 (.32)^a^	49.7 (.73)^a^	57.6 (1.77)^b^	8.8**	.016
Extraversion	48.5 (.28)	50.0 (.63)	50.7 (1.54)	2.7	.005
Openness	46.1 (.28)	46.7 (.63)	45.1 (1.54)	0.7	.001
Agreeableness	50.8 (.31)	49.9 (.69)	49.2 (1.69)	1.1	.002
Conscientiousness	49.2 (.32)^a^	46.8 (.73)^b^	40.3 (1.77)^c^	14.7**	.027
					
N1: Anxiety	51.0 (.29)^a^	49.5 (.66)^b^	54.9 (1.62)^c^	5.6**	.010
N2: Angry Hostility	50.4 (.31)^a^	51.4 (.71)^a^	57.9 (1.73)^b^	9.1**	.017
N3: Depression	50.8 (.36)^a^	51.0 (.82)^a^	58.5 (1.99)^b^	7.2**	.013
N4: Self-consciousness	49.6 (.32)^a^	48.5 (.74)^a^	54.4 (1.80)^b^	4.9**	.009
N5: Impulsiveness	49.0 (.30)^a^	50.0 (.69)^a^	54.0 (1.68)^b^	4.7**	.009
N6: Vulnerability	50.5 (.36)^a^	50.2 (.82)^a^	61.3 (2.01)^b^	14.4**	.027
E1: Warmth	48.1 (.32)	48.7 (.74)	49.3 (1.80)	0.4	.001
E2: Gregariousness	50.3 (.31)	51.2 (.71)	49.4 (1.74)	0.9	.002
E3: Assertiveness	49.6 (.32)	50.0 (.73)	47.3 (1.78)	1.0	.002
E4: Activity	47.3 (.30)	48.5 (.69)	46.2 (1.69)	1.7	.003
E5: Excitement-Seeking	47.2 (.29)^a^	50.0 (.66)^b^	51.0 (1.62)^b^	8.7**	.016
E6: Positive Emotions	48.4 (.32)	48.0 (.73)	45.7 (1.79)	1.2	.002
O1: Fantasy	48.1 (.28)	48.7 (.63)	48.9 (1.53)	0.5	.001
O2: Aesthetics	48.9 (.30)	49.6 (.69)	50.6 (1.68)	0.8	.002
O3: Feelings	47.0 (.29)	47.3 (.66)	48.7 (1.61)	0.5	.001
O4: Actions	45.5 (.31)^a^	47.5 (.71)^b^	42.3 (1.74)^a^	5.4**	.010
O5: Ideas	47.2 (.31)	46.8 (.69)	47.6 (1.69)	0.2	.000
O6: Values	44.7 (.28)^a^	46.9 (.64)^b^	45.3 (1.55)^ab^	4.8**	.009
A1: Trust	47.1 (.33)^a^	45.4 (.74)^b^	42.6 (1.82)^b^	4.5*	.008
A2: Straightforwardness	50.9 (.31)^a^	50.2 (.70)^a^	46.5 (1.72)^b^	3.3*	.006
A3: Altruism	50.6 (.32)	50.7 (.73)	52.1 (1.79)	0.4	.001
A4: Compliance	49.4 (.34)^a^	49.0 (.78)	45.3 (1.91)	2.3	.004
A5: Modesty	51.2 (.32)	51.2 (.72)	52.2 (1.75)	0.2	.000
A6: Tender-mindedness	52.2 (.31)	52.5 (.71)	52.1 (1.72)	0.1	.000
C1: Competence	49.7 (.35)^a^	47.4 (.79)^b^	37.0 (1.92)^c^	22.3**	.041
C2: Order	47.2 (.29)	47.1 (.67)	44.3 (1.63)	1.5	.003
C3: Dutifulness	48.3 (.32)^a^	46.4 (.73)^b^	42.5 (1.77)^c^	7.2**	.014
C4: Achievement Striving	48.5 (.34)^a^	46.7 (.77)^b^	38.1 (1.87)^c^	15.9**	.029
C5: Self-Discipline	48.1 (.34)^a^	46.1 (.78)^b^	39.0 (1.91)^c^	12.6**	.023
C6: Deliberation	52.7 (.33)^a^	51.0 (.75)^a^	42.1 (1.84)^b^	16.6**	.031

*Note*. Estimated marginal means (Standard Errors), statistical tests, and effect sizes computed after controlling for age, sex, ethnicity (black, white), and education (*n *= 1062).

Means with different superscripts differ significantly at *p *< .05.

* *p *< .05; ** *p *< .01

## Discussion

The associations observed in the Baltimore ECA sample are consistent with the existing literature, which finds drug users generally high on measures of negative emotionality or psychopathology and low on Conscientiousness [8,9,20,37]. Most previous studies have analyzed a limited number of traits (often omitting the crucial Conscientiousness factor) or focused on a single substance, making it difficult to integrate the body of evidence across traits or substances. Using a more integrative approach, this study indicates that low Conscientiousness and high Neuroticism are consistently associated with tobacco smoking, heroin, and cocaine use. Low Conscientiousness is also characteristic of marijuana users, who are average on Neuroticism and high on Openness, a trait that distinguishes marijuana users from other drug users. The present study extends the previous literature by assessing higher-level personality factors as well as lower-level facets. The association among Neuroticism and tobacco/heroin/cocaine use was found for all six facets of Neuroticism, indicating that multiple aspects of negative emotionality and psychopathology are involved in this effect. With the exception of C2:Order, low scores on all facets of Conscientiousness were associated with drug use. Although Extraversion showed no association with drug use on the factor level, facet-level analyses revealed a consistent association between high scores on E5:Excitement-Seeking and all types of drug use. This finding is not surprising given that, together with N5:Impulsiveness (inability to resist cravings), C5:Self-Discipline (limited ability to stay on task), and C6:Deliberation (lack of careful consideration of the consequences of one's actions), the E5:Excitement-Seeking facet is an aspect of impulsivity [36]. The selective association between drug use and this specific Extraversion facet also suggests that inconsistent findings for Extraversion may be due to Extraversion measures that differ in their relative emphasis on the excitement-seeking component.

Cross-sectional association analyses provide limited input on the cause and effect relation between personality traits and drug use. Although individual differences in personality traits are particularly stable in adulthood [38-40], some evidence suggests that substance use influences personality-related variables [41]. Cigarette smoking contributes to stress, negative affect states, and the onset of clinical correlates of Neuroticism, such as anxiety and depressive disorders [42-44]. Piedmont [45] reported substantial declines in Neuroticism and increases in Agreeableness and Conscientiousness in a group of polysubstance abusers following a rehabilitation program [26] (but see [28]). Consistently, smoking cessation is associated with lower Neuroticism scores, a lower level of stress, and lower risk of anxiety disorders [42,44]. Another set of evidence suggests that personality traits are risk factors for psychoactive substance use, along with social environment and life experiences [46,47]. For example, in long term longitudinal studies, low Conscientiousness in childhood predicts cigarette smoking in adulthood [48,49]. Longitudinal studies in Europe also suggest that high scores on Neuroticism and Extraversion during adolescence increase the likelihood of being a smoker later in life [18,50]. A common hypothesis is that individuals with high Neuroticism use drugs to self-medicate [51,52]. Finally, third variables might be responsible for the association of personality and addictive behaviors. For example, personality traits and cigarette smoking are both highly heritable [53,54], and could be influenced by common genetic factors [37].

## Limitations

There are several limitations to consider when interpreting the results. This sample is not representative of the entire US population, but it was drawn from a probability sample that included a wide range of socio-economic conditions. There may be some misclassification with the categories of never, former, and current-users. For example, some individuals might be reluctant to disclose their illicit drug use. Some might not recall use in the distant past. Categorizing current users based on self-reported behavior during the past year might be too broad. There are marked differences in the frequency and quantity of drug use, but the relatively small number of users in the present sample does not allow finer distinctions or the use of stricter criteria of addiction. However, preliminary analyses using different classification criteria produced similar results, and the main findings are mostly consistent with the literature. In addition to self-report ratings, future studies should use multiple methods for assessing drug use and personality traits.

Most studies on drug use are conducted in adolescents and young adults, who are at life stages associated with the greater use of drugs. We presented data from an older cohort, which has presumably passed the experimentation age. While this contributes to the scarce literature on drug use in later parts of the lifespan, the advantages of a lifespan perspective come at the cost of having fewer current users in this older cohort. In addition, older cohorts only include the survivors among those who started drug use early in life, which may introduce attrition and other biases. A review of the literature [20] suggested that studies that involve older populations (over the age of 30 years) report weaker association of Conscientiousness-related traits and drug use.

Finally, some of the findings may be culture-bound [55]. For example, the results for smoking closely replicate the findings we previously reported from another US cohort of similar age but different socio-economic status. However, studies conducted in Europe [50] and Japan [17] have found Extraversion associated with cigarette smoking [16]. Such differences might reflect the different social acceptance of smoking across countries.

## Conclusions: Clinical and social policy implications

Personality traits are associated with the outcome of therapeutic interventions. For example, several studies found Neuroticism, anxiety, and depressive disorders related to poor treatment outcome for nicotine dependence [56]. Although we found systematic differences between the personality profiles of substance users and non-users, there is substantial variability in both groups (e.g., not all smokers score high on Neuroticism or low on Conscientiousness). Individual differences among substance abusers can play an important role in the choice of treatment options [57]. Recently, more attention has been focused on personality trait effects on the efficacy of different treatment plans [58] to tailor therapeutic interventions to individual needs [59,60]. More research is needed to fully evaluate how personality assessment can be useful in the choice of treatment plans.

Although individual treatments might reduce the rate of drug abuse, public policy is an important tool for cigarette smoking and other drug abuse prevention and cessation. Because of the low conscientiousness, high impulsivity, and high emotional vulnerability of most drug users, relying on an individual's resources, without therapeutic intervention, may produce limited results. Evidence-based interventions such as safer injecting environments are an important adjunct which can reduce drug-related harm [61]. In the case of cigarette smoking, societal pressure in the form of high taxation, restriction in advertising, and interdiction of smoking in public places are cost-effective programs that are reducing the prevalence of smoking [62].

## Competing interests

Paul T. Costa, Jr. receives royalties from the Revised NEO Personality Inventory. The authors declare that they have no other competing interests.

## Authors' contributions

The ECA is an ongoing longitudinal study in which PTC, OJB, and RMC are active research members that participate in its conception, design, and coordination. AT conceived the current manuscript, performed the statistical analysis, and drafted the manuscript. CEL, PTC, RMC, and OJB contributed to the conception and draft of the manuscript. PTC coordinated the work for the manuscript. All authors read and approved the final manuscript.

## Pre-publication history

The pre-publication history for this paper can be accessed here:


